# Improved islet recovery and efficacy through co-culture and co-transplantation of islets with human adipose-derived mesenchymal stem cells

**DOI:** 10.1371/journal.pone.0206449

**Published:** 2018-11-12

**Authors:** Anissa Gamble, Rena Pawlick, Andrew R. Pepper, Antonio Bruni, Adetola Adesida, Peter A. Senior, Gregory S. Korbutt, A. M. James Shapiro

**Affiliations:** 1 Alberta Diabetes Institute, University of Alberta, Edmonton, AB, Canada; 2 Department of Surgery, University of Alberta, Edmonton, Alberta, Canada; 3 Members of the Canadian National Transplant Research Project (CNTRP), Edmonton, AB, Canada; 4 Clinical Islet Transplant Program, University of Alberta, Edmonton, AB, Canada; 5 Department of Medicine, University of Alberta, Edmonton, Alberta, Canada; University of Toledo, UNITED STATES

## Abstract

Islet transplantation is an established clinical procedure for select patients with type 1 diabetes and severe hypoglycemia to stabilize glycemic control. Post-transplant, substantial beta cell mass is lost, necessitating multiple donors to maintain euglycemia. A potential strategy to augment islet engraftment is the co-transplantation of islets with multipotent mesenchymal stem cells to capitalize upon their pro-angiogenic and anti-inflammatory properties. Herein, we examine the *in vitro* and *in vivo* effect of co-culturing murine islets with human adipose-derived mesenchymal stem cells (Ad-MSCs). Islets co-cultured with Ad-MSCs for 48 hours had decreased cell death, superior viability as measured by membrane integrity, improved glucose stimulated insulin secretion and reduced apoptosis compared to control islets. These observations were recapitulated with human islets, albeit tested in a limited capacity. Recipients of marginal mouse islet mass grafts, co-transplanted with Ad-MSCs without a co-culture period, did not reverse to normoglycemia as efficiently as islets alone. However, utilizing a 48-hour co-culture period, marginal mouse islets grafts with Ad-MSCs achieved a superior percent euglycemia rate when compared to islets cultured and transplanted alone. A co-culture period of human islets with human Ad-MSCs may have a clinical benefit improving engraftment outcomes.

## Introduction

Islet transplantation is a therapeutic procedure that can restore endogenous insulin production and maintain euglycemia for a sustained period in patients with difficult to control type 1 diabetes mellitus (T1DM). The recent Clinical Islet Transplant Consortium’s, National Institute of Health (NIH) sponsored phase 3 trial demonstrated islet transplantation’s ability to stabilize glycemic control in select patients with T1DM presenting hypoglycemia unawareness where the primary end-point revealed 88% and 71% of recipients maintained euglycemia for 1-year and 2-years post-islet transplant respectively [1]. This Federal Drug Administration (FDA)-Biologics License Application enabling study may allow product licensure for islet transplant, facilitating reimbursement through insurance in the USA. Despite its apparent success, this procedure is not without limitations. A major challenge is overcoming suboptimal acute engraftment, where up to 60% of the initial transplanted islet mass is potentially lost due to innate instant blood-mediated inflammatory reaction (IBMIR), delayed re-vascularization, or hypoxic stress [2–5]. Multiple islet infusions are therefore often required to maintain periods of insulin independence. Co-transplantation of islets with multipotent stem cells (MSCs) is a potential strategy to mitigate early islet cell loss in culture and after transplantation [6, 7]. MSCs are ubiquitous throughout cell types, and their capacity for self-renewal and differentiation into cells of mesoderm lineage includes adipocytes, chondrocytes, osteoblast and myocytes [8]. Previous studies have demonstrated the ability of MSCs to augment islet function, in part due to MSC’s immunomodulatory and trophic properties, and their ability to secrete several paracrine factors [9–11]. Notably, MSCs modulate angiogenesis through gene expression of cytokines, including vascular endothelial growth factor (VEGF), fibroblast growth factors (FGFs), transforming growth factor-βs (TGF-βs) and Annexin-1 (ANXA1) [12–14]. MSCs can modulate the secretion of cytokines and promote the concentration of growth factors at the islet engraftment site and may aid neovascularization [15, 16].

In the clinical islet allograft setting, islets undergo an obligate culture period of up to 72 hours before transplantation. The culture period facilitates recipient conditioning, and may allow for transplantation of a more immunologically quiescent graft [17, 18]. Conversely, culture may be detrimental to islet survival due to limited nutrients, and exposure to oxidative, hypoxic, and inflammatory stressors [19]. These stressors lead to impaired islet viability and decreased cell mass. In addition, islet endothelial cells are compromised during the islet isolation and culture process that diminishes islet recovery and function prior to transplantation [20, 21]. During isolation, islets are stripped of their native vascularization and rely on diffusion of nutrients and oxygen to survive. Moreover, the intra-islet endothelial cells rapidly decline to 5% by 4 days post culture [20]. The disrupted vascular supply hinders the post-transplant revascularization process. Over this period, insufficient vascularization causes increased cell death and graft failure due to inadequate nutrient delivery and prolonged ischemia [21]. In consequence, multiple donors and infusions are often required to maintain recipient insulin independence [19, 22, 23]. To decrease donor demand, it has been shown co-culturing islets with human bone marrow-derived MSCs or mouse adipose-derived MSCs (Ad-MSCs), for an extended period, can augment islet function and improve islet engraftment post-transplant [24–26].

In the present study, we explored the use of human Ad-MSCs co-cultured with murine and human islets for 48 hours at islet to Ad-MSC ratios of 1:300 [24] and 1:2000 [25]. We hypothesize co-culturing islets with human Ad-MSCs will improve islet recovery, islet function and augment engraftment outcomes.

## Materials and methods

### Murine pancreatectomy and islet solation

Pancreatic islets were isolated from 8- to 12-week-old BALB/c mice (Jackson Laboratories, CA) and housed under conventional conditions in accordance with the Canadian Council on Animal Care. All experimental procedures were approved by the University of Alberta Research Ethics and Animal Use Committee (Study ID: AUP00000331). Prior to pancreatectomy, the common bile duct was cannulated and the pancreas was distended with 0.125 mg/mL cold Liberase TL Research Grade enzyme (Roche Diagnostics, Laval, QC, CA) in Hanks balanced salt solution (Sigma, St. Louis, MO, USA). Pancreas digestion was continued in a 37°C water bath for 14 minutes with light agitation. After the pancreatic digestion phase, islets were purified using histopaque-density gradient centrifugation (1.108, 1.083 and 1.069 g/mL, Sigma, St. Louis, MO, USA). Upon purification, islets were placed in Connaught Medical Research Laboratories (CMRL-1066) (Corning-cellgro, Manassas, VA, USA) supplemented with 10% fetal bovine serum, 1% L-glutamine (200 mmol/L, Sigma, St. Louis, MO, USA), 1% sodium pyruvate (100 mmol/L, Sigma, St. Louis, MO, USA), 1% non-essential amino acid 100x (Sigma, St. Louis, MO, USA) and 100 U/mL penicillin-G and100 μg/mL streptomycin (Sigma Aldrich Canada Co., Oakville, ON, CA) at 37°C/5%CO_2_ at pH 7.4.

### Human islet isolation, purification and culture

Human islet preparations were isolated from deceased consenting multi-organ deceased donors, as described [19], with intent for clinical transplantation. Islets were only used for research when yields failed to meet that required for a listed recipient and with research consent from donor families. Permission for these studies was granted by the University of Alberta Health Research Ethics Board (REB), Edmonton, Alberta, Canada. The REB ensures that individual research projects involving human participants, identifiable data and/or human biological material meet the requirements of the current *Tri-Council Policy Statement*: *Ethical Conduct of Research Involving Humans* and University policy as well as provincial, federal and other legislation and regulations, as applicable.

### Adipose derived mesenchymal stem cell expansion and culture conditions

To prepare the human adipose-derived MSCs, infrapatellar fat pad were removed from 2 donors (18 and 25 year old males) undergoing orthopedic knee surgery at the University Hospital of Alberta, Edmonton, Canada, and processed as described before [27]. The Ad-MSCs meet the International Society for Cellular Therapy guidelines [28] and were verified through chondrogenic differentiation after stimulation with TGFbeta and dexamethasone and CD cell surface markers [27]. Ethics committee waived the need for written informed consent of patients, as specimens used in this study were intended for discard in the normal course of the surgical procedure. All research involving human participants were reviewed and approved by the University of Alberta Health REB (Study ID: PRO 00001416 and PRO 000018778).

Cryopreserved Ad-MSCs containing 2x10^6^ cells per vial were thawed upon second passage and population doubling of 4. For expansion, cells were plated in flasks containing Eagle’s minimum essential medium (MEM) (Sigma Aldrich Canada Co., Oakville, ON, CA) supplemented with 2.5 ng/mL basic fibroblast growth factor (Millipore, Etobicoke, ON, Canada), 10% fetal bovine serum, L-glutamine (2 mmol/L) (Sigma, St. Louis, MO, USA), penicillin (50 000 units) and streptomycin (50 mg) (Sigma Aldrich Canada Co., Oakville, ON, CA), HEPES (5 mmol/L) and sodium pyruvate (5 mmol/L) (Sigma, St. Louis, MO, USA) at 37°C/5%CO_2_ and a pH 7.4. The medium was changed within the first 24-hours, and thereafter in 48-hours intervals. Once confluent, the cell monolayer was washed with Versene (1% EDTA in PBS; Life technologies Inc. Burlington, ON, Canada) and enzymatically detached with 0.5% trypsin-EDTA. Cells were counted and aliquoted (4 x10^5^ cells) onto 60 mm × 15 mm ultra-low adherence culture dishes (Corning, Corning, NY, USA). All samples were cultured in suspension, left to gravity settle under static conditions without agitation, stirring or mixing throughout the 48 hours of culture. Experimental groups contained 200 BALB/c islets, and all groups (islets alone, islet: Ad-MSCs 1:300 and 1:2000) contained 5 mL of CMRL culture media for 48 hours. The MSCs isolated abided by the Mesenchymal and Tissue Stem Cell Committee of the International Society for Cellular Therapy guidelines.

### Percent islet recovery

Post isolation and subsequently post 48-hour culture, islets were harvested and counted to determine islet yield. Aliquots from respective samples were stained with dithizone (Sigma Aldrich Canada Co., Oakville, ON, CA) and counted in triplet. The percentage of islet recovery was determined by ratio of total islets harvested 48 hours’ post-culture relative to the number of islets harvested immediately post-isolation.

### Insulin secretory activity

Following isolation and post 48-hour culture, triplicates of 50 mouse and human islet equivalents were collected for respective groups and a static glucose-stimulated insulin secretion (s-GSIS) assay and dynamic insulin perfusion was performed. Islets were washed of residual glucose in glucose free medium, followed by incubation in RPMI-1640 (Sigma Aldrich Canada Co., Oakville, ON, CA) containing low (2.8 mmol/L) glucose, followed by high (16.7 mmol/L) glucose, each, for one hour at 37ºC. The supernatant was harvested and stored at 20ºC. Insulin levels were measured by an enzyme-linked immunosorbent assay (ELISA) (Mercodia, Uppsala, Sweden).

Dynamic insulin perifusion [29] was assessed by an automated perifusion system (Bio rep® Perifusion). Islets were exposed to Krebs solution containing low glucose (2.8 mmol/L, 8–16 mins), high glucose (28 mmol/L, 17–30 mins) and KCL solution (20 mmol/L KCl in 2.8 mmol/L glucose, 31–45 mins) for respective time periods. The perfusate was collected in an automated multiwell plate format and stored at -20°C until analyzed for insulin quantification by an ELISA (Alpco, Salem, NH, USA).

### Apoptosis TUNEL staining

Apoptosis of islets was assessed quantitatively using Tdt-mediated dUTP nick-end labeling (TUNEL) staining. Prior and post-cultured islets were fixed in 4% paraformaldehyde, embedded in agar, processed and embedded in paraffin. Tissue sections were co-stained with anti-insulin antibody at 1:200 concentration (Agilent Technologies, Mississauga, ON, CA) and labeled with Rhodamine (TRITC) conjugated anti-guinea pig IgG (1:200, Jackson ImmunoResearch, West Grove, PA, USA). To identify the apoptosis, fluorescein isothiocyanate-dUTP with TdT enzyme (Promega, Madison, WI, USA) was added and counterstained with the nuclear stain, DAPI (ProLong Gold DAPI, Invitrogen, Calrsbadm CA, USA). Apoptosis was determined by analyzing the number of positive TUNEL-stained cells as a percentage of both insulin and nuclei positive cells utilizing FIJI ImageJ Software (National Institute of Health, USA).

### Mesenchymal stem/stromal cell tracking

Following isolation, MSCs were stained with a vybrant DiO cell labeling dye (ThermoFisher Scientific, Waltham, MA) dosed at 5μl per 10^6^ cells. Stained islets were distributed amongst respective groups of MSCs alone, 1:300 and 1:2000 islet to MSC ratios. Islets cultured alone did not contain MSCs. Microscopic evaluation was performed prior to culture (0 hours) and post culture (48 hours). MSCs microscopic evaluation and quantification was performed by FIJI ImageJ Software (National Institute of Health, USA).

### Pro-inflammatory cytokine assessment

Pro-inflammatory cytokines were analyzed from culture media post 48-hour culture. Media was assessed for mouse tumor necrosis factor (TNF)-α, KC-GRO, interferon (IFN)-γ, interleukin (IL)-1β, IL-10, IL-6 and IL-12p70 using a Mouse ProInflammatory 7-Plex Tissue Culture Kit (Meso Scale Diagnostics, Rockville, Maryland, USA). Human IL-1β, IL-12p70, IL-6, IL-8, IL-10, IL-13, TNF-α and IFN-γ cytokine analysis was demonstrated using a Human ProInflammatory Panel kit (Meso Scale Diagnostics, Rockville, Maryland, USA). The plates were loaded into an MSD-SECTOR® instrument for electrochemiluminescence analysis.

### Islet transplantation

To induce diabetes, female and male adult (8–10 weeks, 20–30 gm) immunodeficient mice (B6.129S7-Rag1^tm1Mom/J^; Jackson Laboratories, CA) were administered an intraperitoneal injection of streptozotocin (175mg/kg i.p) (Sigma, St. Louis, MO, USA) in acetate phosphate buffer (Sigma Aldrich Canada Co., Oakville, ON, CA) at pH 4.5. Mice with blood glucose levels ≥18 mmol/L for two daily consecutive non-fasting blood glucose readings, were considered diabetic.

The mice were transplanted with post-isolation or post-culture mouse islets with or without the presence of Ad-MSCs (islet to Ad-MSC ratio: 1:2000) or islets alone. Islets were aspirated into polyethylene (PE-50) tubing and centrifuged. A left lateral paralumbar subcostal incision was made and islets were delivered under the left kidney capsule. Islet engraftment was assessed through non-fasting blood glucose measurements, three times per week for 60 days. Blood glucose monitoring was conducted using a portable glucometer (OneTouch Ultra 2, LifeScan, CA, USA). Two consecutive readings maintained at <11.1 mmol/L confirmed graft function and reversal of diabetes. At day 60, a recovery nephrectomy of the grafted kidney confirmed graft dependent efficacy, when animals returned to hyperglycemia (≥18 mmol/L).

### Intraperitoneal glucose tolerance test (IPGTT)

*In vivo* glucose tolerance and islet function was assessed by an intraperitoneal glucose tolerance test (IPGTT) in euglycemic mice 6 weeks’ post-transplant. The mice were fasted overnight and administered 25% dextrose intraperitoneally at a dose of 3 g/kg. Naïve normoglycemic mice served as controls. Blood glucose was monitored at baseline (t = 0), 15, 30, 60, 90 and 120 minutes’ post injection. Significance was measured as area under the curve amongst groups.

### Islet graft assessment

Sixty days’ post islet transplant, kidney bearing grafts were removed and placed in -80°C. Kidneys were homogenized and sonicated with 2mM of acetic acid in 0.5% of bovine serum albumin (Thermo Fisher Scientific, Waltham, MA, USA). Supernatant was collected and insulin levels were measured by an enzyme-linked immunosorbent assay (ELISA) (Mercodia, Uppsala, Sweden).

Immunohistochemistry was performed on a subset of kidneys bearing the islet grafts post 60 days’ post islet transplant and which were fixed in 10% formalin. The tissue was sectioned and upon heat retrieval, were blocked with 20% goat serum (Sigma-Aldrich, St. Louis, MI, USA), and subsequently incubated overnight at 4°C primary antibodies guinea pig anti-insulin (1:200, Dako, Mississauga, ON, Canada) and rabbit anti-glucagon (1:100, Abcam, Cambridge, MA, USA). The following day sections were washed and incubated with secondary antibodies for 1 hour at room temperature utilizing goat anti-guinea pig Rhodamine and goat anti-rat fluorescein (1:200 Jackson ImmunoResearch, West Grove, PA, USA) counterstained with DAPI (ProLong Gold DAPI, Invitrogen, Calrsbadm CA, USA).

### Statistical analysis

Data were analyzed using GraphPad Prism (GraphPad Software, La Jolla, CA, USA). Student *t* test or analysis of variance (ANOVA) was used to compare islet yield, membrane integrity, TUNEL, sGSIS and AUC for IPGTT’s and represented as scatter plots. Kaplan-Meier graft survival function curves were compared using the log-rank (Mantel-Cox) statistical method. Statistical significance was considered when p-values < 0.05. Graphical representation of data is as mean ± standard deviation (s.d) of small *in vitro* data sets, and standard error of the mean (s.e.m) for *in vivo* analysis where *p < 0.05, **p < 0.01, ***p < 0.001, ****p < 0.0001.

## Results

### Mouse islets co-cultured with human adipocyte derived mesenchymal stem cells improve *in vitro* function

**Fig 1:**
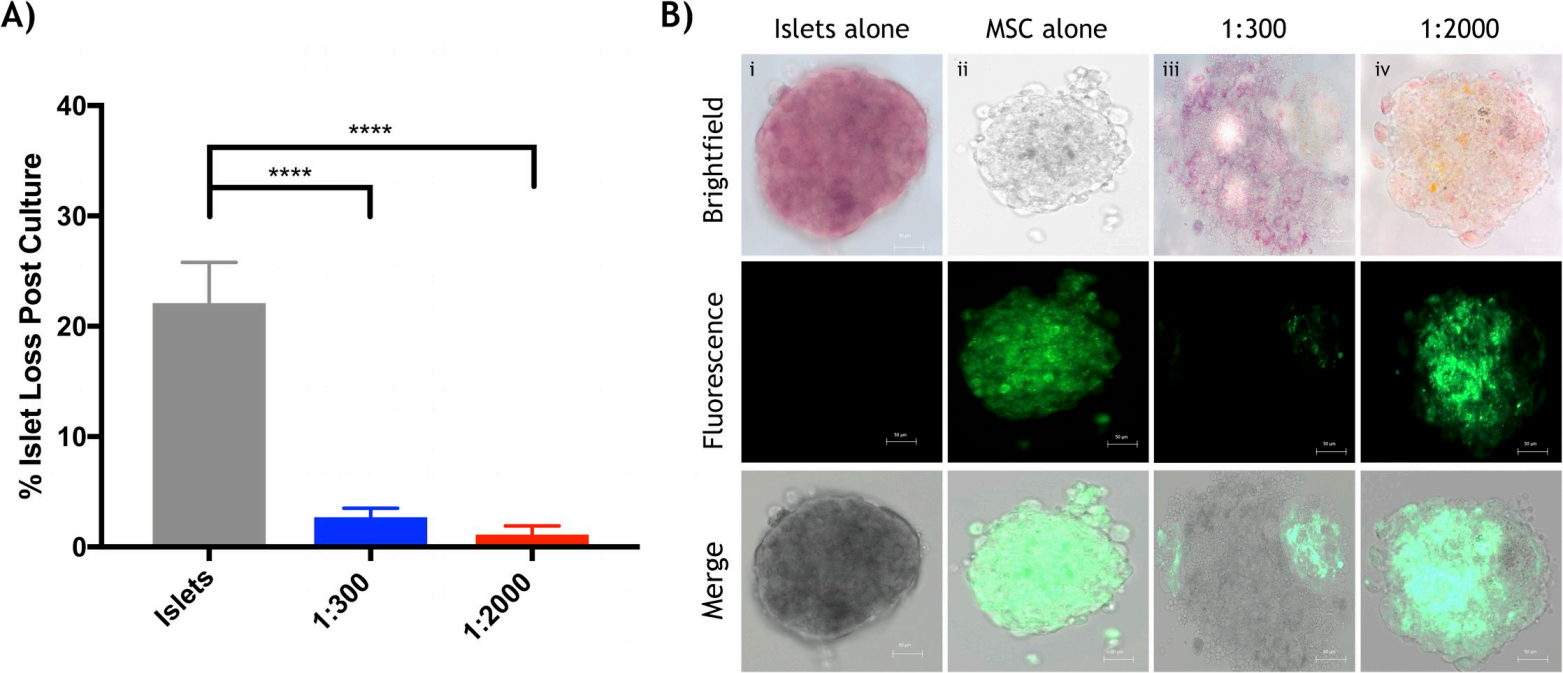
Quantitative and qualitative analysis of human adipose derived mesenchymal stem cells co-cultured with murine islets. *In vitro* assessment of experimental groups post 48-hour co-culture period. (a) The percentage of islet cell loss post 48-hour co-culture for islets cultured alone (grey) was significantly less than both the islet:Ad-MSCs at 1:300 (blue) (****p<0.0001, ANOVA) and 1:2000 (red) (****p<0.0001, ANOVA). (b) Microscopic imaging of labelled human adipose derived mesenchymal stem cells accumulation around mouse islet cells after culture. Column (i) Islets cultured alone, (ii) Ad-MSCs cultured alone, (iii) 1:300 islet to Ad-MSC ratio and (iv) 1:2000 islet to Ad-MSC ratio. Rows (from top to bottom) include bright field microscopic imaging with islets stained with dithizone, fluorescence imaging displaying cell labelled mesenchymal stem cells (DiO staining) displayed in green and combined brightfield and fluorescence microscopic images. The scale bar represents 50μm and data is represented as (mean ± s.e.m).

**Fig 2:**
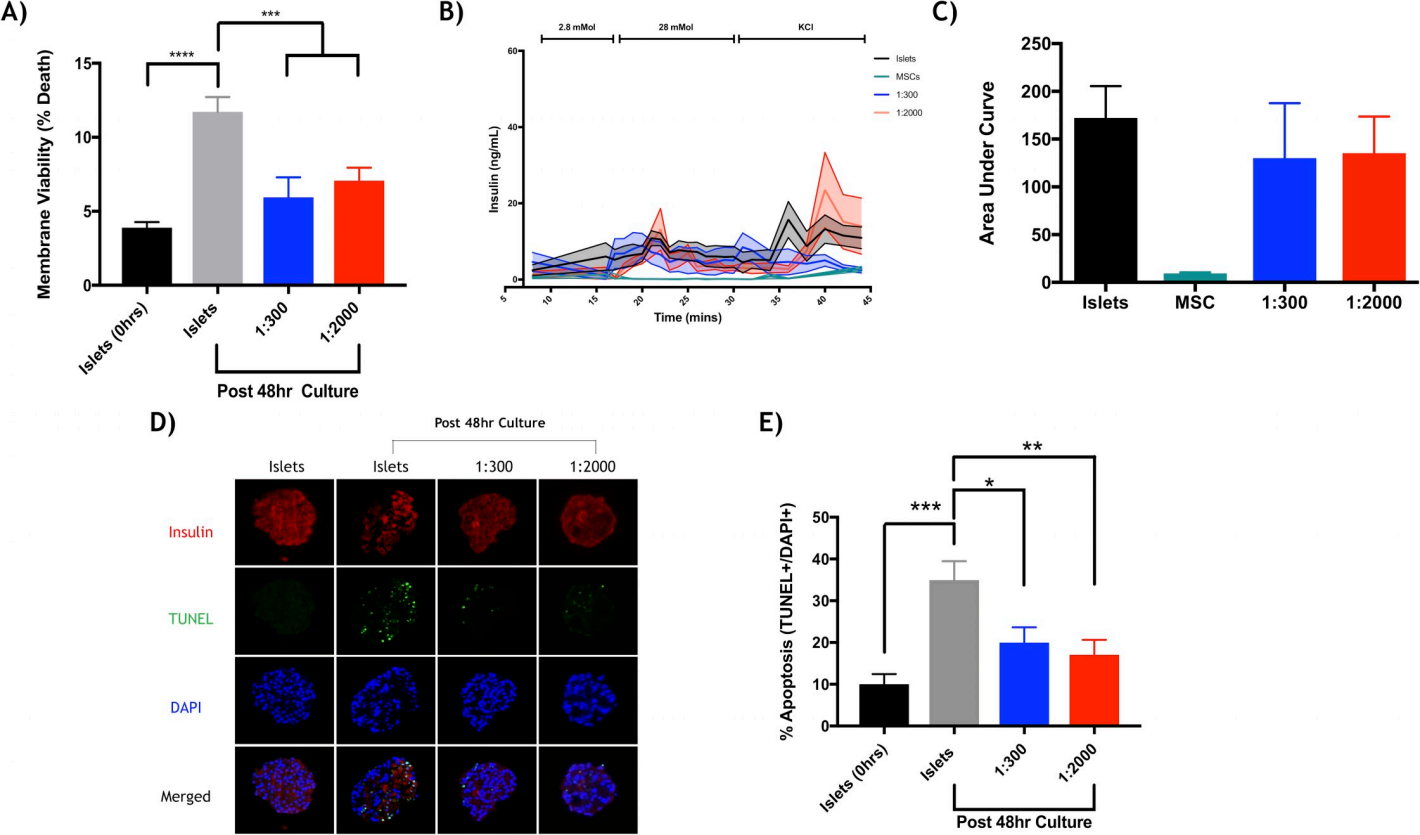
In vitro assessment of mouse islet viability and function after co-culture with adipose mesenchymal cells. (**a**) Islet and Ad-MSC Live/dead staining by dual-fluorescence revealed mouse islets cultured alone (grey) exhibited significantly reduced islet viability compared to when cultured with human Ad-MSCs after 48 hours in culture, islets to Ad-MSCs at 1:300 (blue) and 1:2000 (red) (***p<0.001, ANOVA). There was no significant difference when Ad-MSCs with islets were compared to islets alone immediately after isolation (black). (**b**) A dynamic perifusion assay was also completed after the 48-hour culture period for islets alone (black), mesenchymal stem cells alone (teal), and the combination at 1:300 (blue) and 1:2000 (red). (**c**) Area under the curve for the perifusion assay displayed no significant difference amongst all groups (*p>0.05, ANOVA). (**d**) Cell apoptosis was assessed by TUNEL staining of insulin (red), apoptosis (green) and nucleus/ DAPI (blue). (**e**) Upon analysis, there was significant cell death for islets and Ad-MSCs cultured alone (grey) compared to those cultured with Ad-MSCs at 1:300 (*p<0.05, ANOVA) and 1:2000 (**p<0.01, ANOVA). Apoptosis percentage was analyzed by FIJI software by the surface area of TUNEL positive over DAPI positive. Data represented as (mean ± s.e.m).

Murine islets co-cultured with human Ad-MSCs improved islet recovery, viability, survival and function. After 48-hour of co-culture, islets were quantified to determine islet yield. Ad-MSCs improved islet yield (**Fig 1A**) and demonstrated ability to adhere to the islet surface (**Fig 1B**). Control islets cultured alone exhibited significant islet loss (22.1 ± 10.5% islet loss) compared to both co-cultured Ad-MSC groups: 1:300 (2.7 ± 1.9%) and 1:2000 (1.1 ± 0.81%) (p<0.0001 respectively). Dual-fluorescence staining assessing islet viability revealed islets co-cultured with Ad-MSCs, 1:300 (5.9 ± 1.3%) and 1:2000 (7.1 ± 0.09%), maintained greater viability, less percentage of cell death, relative to islets cultured alone (11.7 ± 0.9%) (p<0.05, p<0.01, respectively, **Fig 2A**). Maximal insulin secretory function was assessed by a static glucose stimulated insulin secretion (sGSIS) assay and revealed islets co-cultured with a 1:2000 islet to Ad-MSC ratio had significantly higher stimulation index relative to islets cultured alone (islets: 0.95 ± 0.15 vs. 1:2000: 3.29 ± 0.76, p<0.05) (**Table 1**). A time challenged dynamic islet perifusion assay to test moment-to-moment dynamic secretory completed after 48-hour culture (**Fig 2B**), illustrated no statistical difference for area under the curve amongst groups (**Fig 2C**). Islets cultured alone had significantly increased apoptosis compared to both Ad-MSC groups. The percentage apoptosis of islets cultured alone was 34.9 ± 4.6% compared to islets cultured with MSCs at 1:300, at 19.9 ± 3.7% vs. islets cultured with MSCs at 1:2000, at 17.0 ± 3.6% (p<0.05 and p<0.001 respectively, **Fig 2D and 2E**).

**Table 1:** Mouse and human static glucose stimulated insulin secretion assay.

	Cellular Insulin			
Group	Condition and Culture Period	2.8 mM Glucose (ng/mL)	16.7 mM Glucose (ng/mL)	Stimulation Index
Mouse Islets Alone	0-hour culture	11.63 ± 1.15†	19.01 ± 1.78* ‖	1.79 ± 0.23
	48-hour culture	2.62 ± 0.57†	2.65 ± 0.43 *§	1.17 ± 0.33 §
Mouse Islets + Ad-MSCs	1:300 48-hour culture	5.07 ± 1.32	9.89 ± 2.03 ‖	1.17 ± 0.33
	1:2000 48-hour culture	6.91 ± 1.81	13.20 ± 2.49 §	2.93 ± 0.85 §
Human Islets Alone	0-hour culture	11.64 ± 3.9	39.30 ± 7.44º ∫	5.13 ± 1.92
	48-hour culture	3.75 ± 1.49	8.29 ± 2.03 º	1.48 ± 0.29
Human Islets + Ad-MSCs	1:300 48-hour culture	10.49 ± 3.96	26.53 ± 6.36	4.36 ± 1.47
	1:2000 48-hour culture	6.67 ± 2.52	17.28 ± 3.29 ∫	4.54 ± 1.15

Data are mean ± s.e.m. of three independent experiments. In each experiment, islets were collected post isolation (0-hour culture) and post 48-hour co-culture, where groups consist of islets alone, islets with 1:300 and 1:2000 islet to human adipose-derived mesenchymal stem cell ratios. Static glucose insulin secretion assay were performed in triplets of 50 islets per group. Stimulation indices were calculated by dividing the amount of insulin released at high glucose (16.7 mM) by that release at low glucose (2.8 mM). Insulin secreted is measured in ng/mL.

* p < 0.0001 mouse islets (0 hrs culture) vs. islets (48 hrs culture)

† p < 0.05 mouse islets (0 hrs culture) vs. islets (48 hrs culture)

‖ p < 0.01 mouse islets (0 hrs culture) vs. islets + 1:300 Ad-MSCs

§ p < 0.05 mouse islets (48 hrs culture) vs. islets + 1:2000 Ad-MSCs

º p < 0,001 human islets (0 hrs culture) vs. islets (48 hrs culture)

∫ p < 0,05 human islets (0 hrs culture) vs. islets + 1:2000 Ad-MSCs

### Pro inflammatory cytokine expression after islet transplant

Post 48-hour culture media was collected and analyzed for murine and human pro-inflammatory cytokines (S1 **Fig**). Murine interleukin (IL)-2p70 expression was reduced by the presence of Ad-MSCs during culture, whereas islets cultured alone revealed increased expression (p<0.05, S1B **Fig**). Human and mouse IL-1β, IL-6, IL-10, and tumor necrosis factor (TNF)-α were detected but their expression amongst respective groups did not provide statistical significance (islets vs. islets +Ad-MSCs) (**S1A, S1C and S1E Fig**).

### Co-transplantation of islets and Ad-MSCs without prior co-culture is detrimental to engraftment

**Fig 3:**
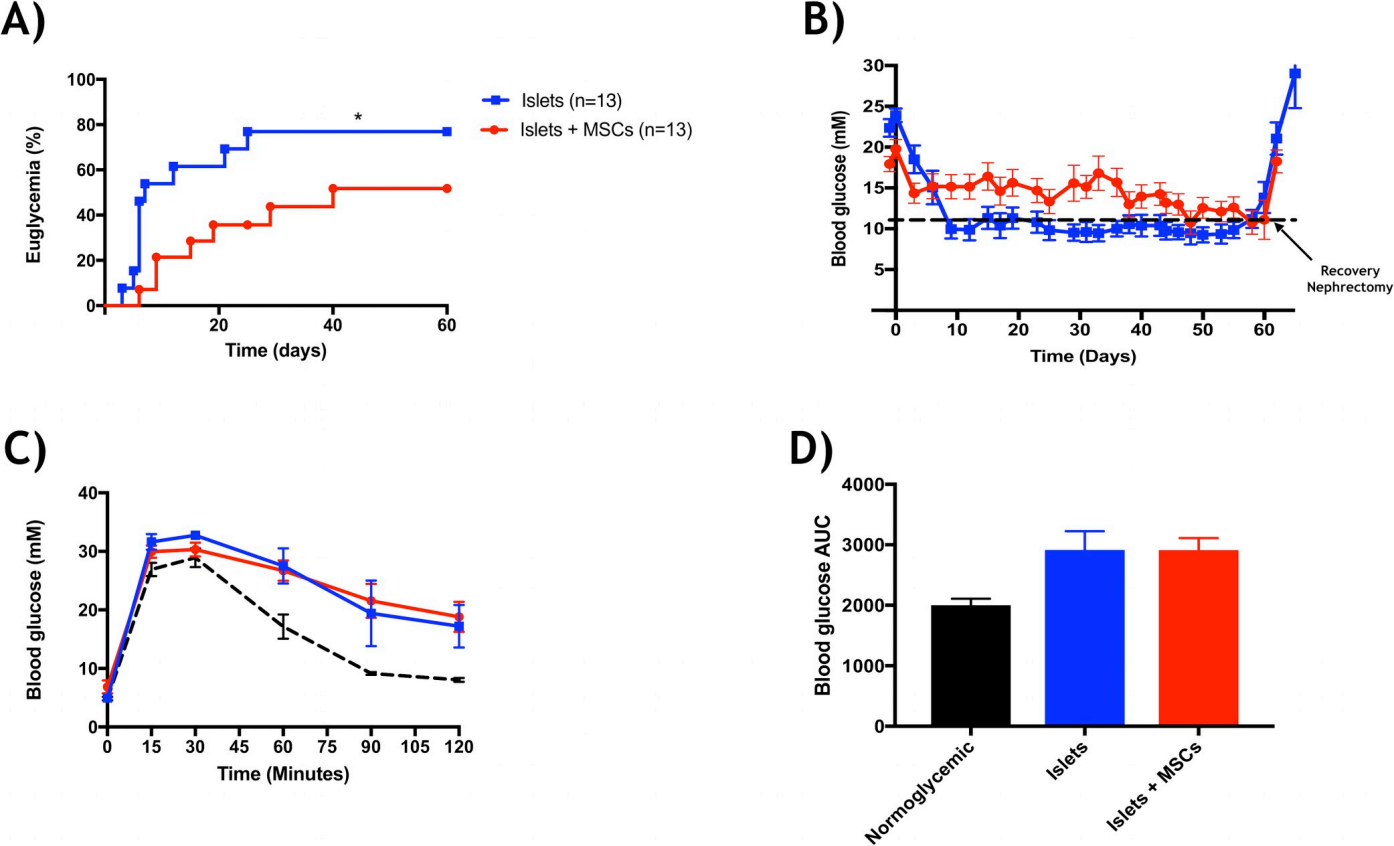
Marginal mass islet co-transplantation and glucose tolerance. Efficacy of a marginal mass (150 BALB/c mouse islets per recipient) under the kidney capsule of diabetic Rag^-/-^ mice with or without the presence of human Ad-MSCs (1:2000). (a) Percent euglycemia exhibited improved diabetes reversal of diabetes for the control group (blue, n = 10 of 13) relative to co-transplant groups (red, n = 7 of 13) (*p<0.05, log-rank). (b) Weekly non-fasting blood glucose measurements, regardless of euglycemia, illustrated improved graft function for islets transplanted alone (blue) relative to islets co-transplanted with Ad-MSCs (red). (c) Intraperitoneal glucose tolerance test (d) and blood glucose area under the curve (AUC) demonstrated a similar trend for respective groups (*p>0.05, ANOVA). Naïve normoglycemic mice served as controls (n = 6) and data represented as (mean ± s.e.m).

To determine if a period of islet-MSC co-culture was needed, we co-transplanted islets + Ad-MSCs without prior culture. One hundred and fifty mouse islets (marginal islet mass) were isolated and co-transplanted under diabetic murine kidney capsule with or without 1:2000 Ad-MSCs combined at the time of transplant. Ten of 13 (76.9%) control islet recipients without MSCs became euglycemic at 21.0 ± 6.4 days after transplant, whereas 7 of 13 (53.8%, p<0.001) islets + Ad-MSCs became euglycemic by 38.6 ± 6.1 days (**Fig 3A and 3B**). All euglycemic animals became hyperglycemic after graft recovery nephrectomy 60 days after transplant, confirming graft dependent euglycemia. Seven weeks’ post-transplant, an intraperitoneal glucose tolerance test (IPGTT) was performed on all euglycemic mice (**Fig 3C**) and demonstrated no significance amongst the mean area under the curve (**Fig 3D**).

### Co-culture for 48 hours of islets and Ad-MSCs followed by co-transplantation improves engraftment

**Fig 4:**
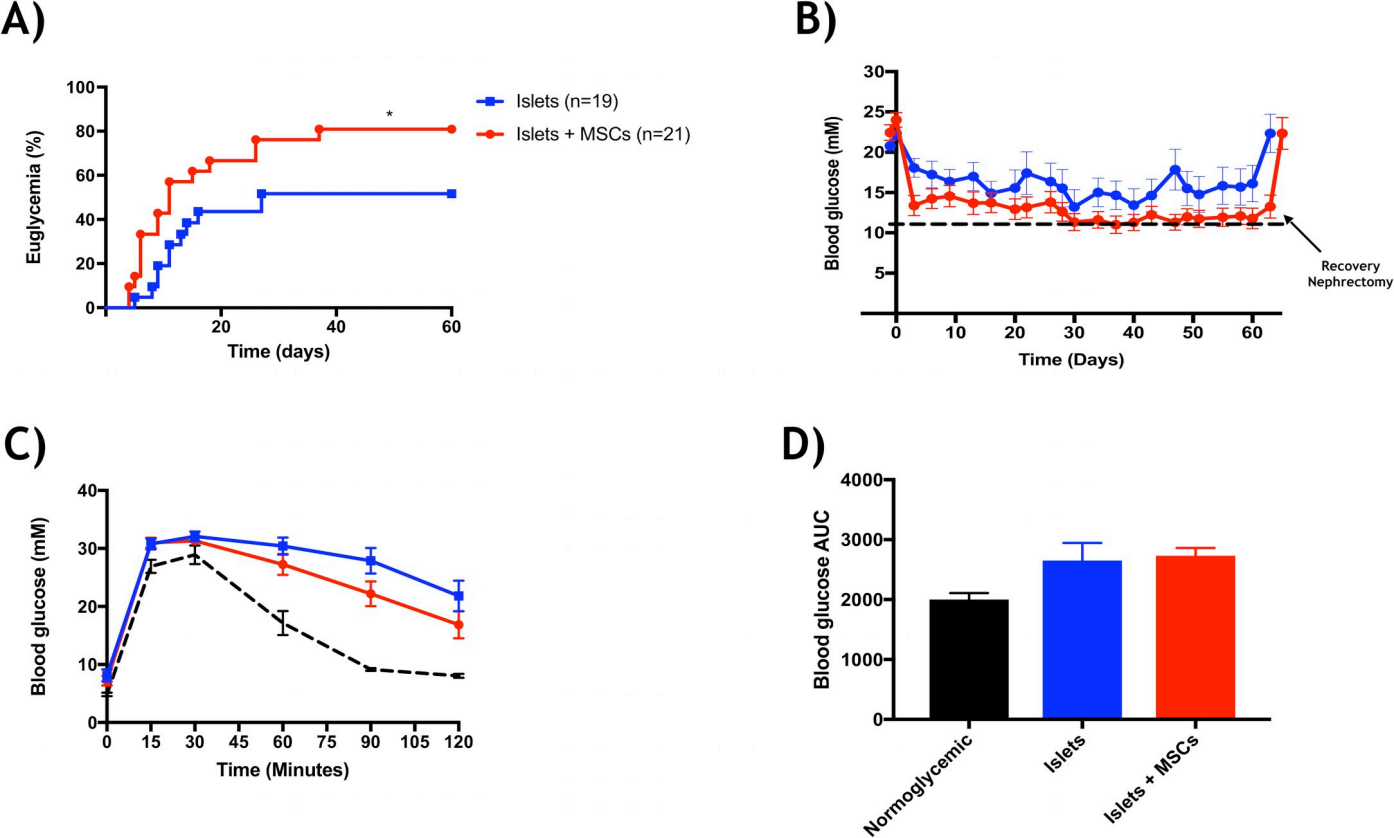
Efficacy of 48-hour co-culture period. Subsequent to the 48-hour co-culture period, a marginal mass of 200 BALB/c islets were counted for respective groups and co-transplanted with human Ad-MSCs under the kidney capsule of diabetic Rag^-/-^ mice. (**a**) Percent euglycemia displayed significant diabetes reversal for the islet-Ad-MSC group, 1:2000 (red, n = 18 of 21) versus the control group (blue, n = 9 of 19) (*p<0.05, log-rank). (**b**) Weekly non-fasting blood glucose measurements, regardless of euglycemia, demonstrated similar glucose profile amongst the control (blue) and 1:2000 (red) groups. (**c**) 7 weeks’ post-transplant an intraperitoneal glucose tolerance test was similar between groups and blood glucose area under the curve (AUC) (**d**) supported no significance amongst respective groups (*p>0.05, ANOVA). Naïve normoglycemic mice served as controls (n = 6) and data represented as (mean ± s.e.m).

Post-isolation, islet aliquots were placed into non-adherent petri dishes and co-cultured with or without the presence of Ad-MSCs for 48 hours (1:2000 islet to Ad-MSC ratio, 48hrs co-culture period) and subsequently transplanted into diabetic mice under the kidney capsule (200 islets per recipient). Mice co-transplanted with Ad-MSCs (1:2000 islet to Ad-MSC ratio) reversed to euglycemia at a faster rate (22.3 ± 4.7 days) than the islets alone group (38.5 ± 7.6 days) (**Fig 4A and 4B**). As well, percent engraftment was improved in those mice transplanted with islets co-cultured with 1:2000 Ad-MSCs (islets: 9 out of 19 (47%) vs. 1:2000: 18 out of 21 (86%)) (p<0.05) (**Fig 4A and 4B**). Sixty days post-transplant, all euglycemic mice returned to hyperglycemia post recovery nephrectomy. IPGTTs were performed on all euglycemic recipients 7 weeks post-transplant (**Fig 4C**) and there was no significant difference for area under the curve amongst respective groups (**Fig 4D**).

### Insulin content of kidney islet engraftment

At 60 days post islet transplant, kidney bearing grafts were removed from both normoglycemic and hyperglycemic mice and measured for insulin content. Islets cultured for 48-hours and co-transplanted did not differ amongst respective groups (islets: 2.1 ± 0.8 ng/g vs. 1:2000: 3.1 ± 1.2 ng/g, S1 **Table**). Immunohistochemistry analyzing the percentage of insulin (islets: 1.8 ± 1.0% vs. 1:2000: 1.4 ± 1.1%) and glucagon (islets: 1.4 ± 0.7% vs. 1:2000: 1.4 ± 0.6%) content did not display significance amongst respective groups (S1 **Table**).

### Human islets co-cultured with mesenchymal cells improve *in vitro* function

**Fig 5:**
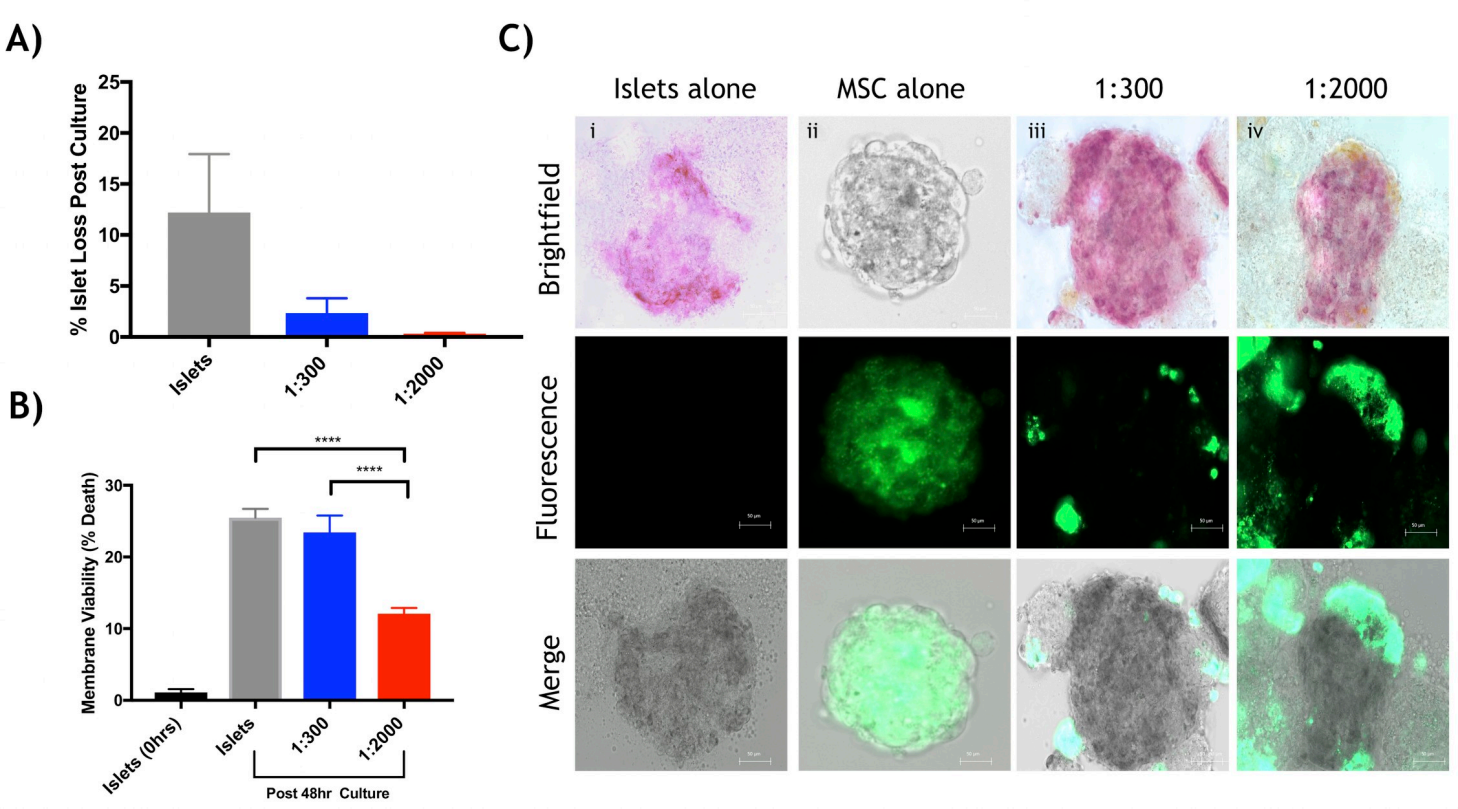
In *Vitro* Assessment of Human Islet Recovery, Viability and Function after Co-Culture with Adipose Mesenchymal Cells. *In vitro* assessment of human islets co-cultured with human adipose derived mesenchymal stem cells (n = 3, isolations). (**a**) The percentage islet loss post 48-hour co-culture was not statistically different amongst groups (p>0.05, ANOVA) (**b**) Live/dead staining by dual-fluorescence revealed human islets cultured with 1:2000 Ad-MSCs (red) had significantly improved islet viability compared to islets cultured alone (grey) and cultured with 1:300 Ad-MSCs (blue) respectively (**p<0.01, ANOVA). (**c**) Microscopic imaging of labelled human adipose derived mesenchymal stem cells adhered to human islet cells after culture. Column (i) Islets cultured alone, (ii) Ad-MSCs cultured alone, (iii) 1:300 islet to Ad-MSC ratio and (iv) 1:2000 islet to Ad-MSC ratio. Rows (from top to bottom) include bright field microscopic imaging with islets stained with dithizone, fluorescence imaging displaying cell labelled mesenchymal stem cells (DiO staining) displayed in green and combined brightfield and fluorescence microscopic images. White bar represents 50μm.

Human islets were examined under the same *in vitro* conditions as mouse islets. Although not significant, human islets cultured alone exhibited increased islet loss (12.2 ± 12.8% islet loss) compared to both Ad-MSC groups: 1:300 (2.3 ± 2.9%) and 1:2000 (0.3 ± 0.2%) (**Fig 5A**). Comparable to mouse islets, Ad-MSCs adhered to human islets (**Fig 5B**). Membrane viability was significantly higher in islets co-cultured with 1:2000 Ad-MSCs (12.1 ± 1.6%) relative to both the 1:300 and islets cultured alone groups (23.5 ± 4.7% and 25.5 ± 2.5%, respectively) (p<0.01, **Fig 5C**). Insulin stimulation index from sGSIS revealed no significant difference amongst groups (islets: 1.3 ± 0.3 vs. 1:300: 3.4 ± 0.9 and 1:2000: 4.5 ± 1.2) (**Table 1).**

## Discussion

In this study, we show that co-culturing mouse islets for 48 hours with human Ad-MSCs improves islet function and efficacy relative to islets cultured alone. Furthermore, we demonstrated at a limited capacity human islets co-cultured with Ad-MSCs may improve *in vitro* islet recovery and function. Collectively, these findings provide a fundamental basis for applications within a clinical setting. Clinical islet transplantation is restricted currently to patients with brittle T1DM that cannot be stabilized by alternative measures. Multiple intraportal islet infusions are often required to sustain euglycemia. Human islet loss during culture and intraportal transplantation is substantial, related in part to IBMIR, hypoxia, apoptosis, and other inflammatory or immune activating events [30–33]. Supplementation of islets with additives during culture may support islet function, morphology, and vitality [34–36]. It has been shown previously that human and mouse islet function can be maintained for up to 30 days after co-culture with MSCs [37, 38]. During the culture period, high doses of mouse derived MSCs and islets adhere to the edges of islets and can penetrate the islet core to form MSC-islet composites [26]. Consequentially, most studies evaluate the utilization of bone marrow derived MSCs and limited research evaluates the use of Ad-MSCs [39, 40]. Studies utilizing Ad-MSCs have shown improved islet function and reduced islet mass to reverse diabetes in rodent models [41–43]. Herein, this study examined the supplementation of mouse or human islets with human-derived Ad-MSCs during a 48-hour co-culture period to evaluate their potential clinical relevance and therapeutic benefits for human islet survival and engraftment.

Our *in vitro* observations demonstrated the ability to increase mouse islet yield, maintain vitality, cell survival and insulin secretion with the supplementation of Ad-MSCs during co-culture relative to islets cultured alone. Our observations are potentially applicable for future translation to the clinic as we demonstrated the capacity of human Ad-MSCs to also improve human islet *in vitro* potency and it can further investigate *in vivo* through the Rag^-/-^ model. Our findings support previous literature where human islets with human-derived Ad-MSCs improved islet function *in vitro* after 72-hour culture on an engineered cell sheet [44] and improved islet efficacy in mice by pre-48-hour-hypoxic cultured human-derived Ad-MSCs intraperitoneally injected into mice [45]. Likewise, rat islets following a 72-hour post-culture with human-derived Ad-MSCs had improved islet engraftment upon intraportal co-transplantation in rats and [46]. Direct static co-culture with MSCs has the potential to release local cytokines, including VEGF and ANAX1, to promote cell survival [13, 14, 47]. We did not measure such trophic factors but in an effort to comprehend and analyze the activity of Ad-MSCs we collected media after 48h co-culture to quantify proinflammatory expression [48–50]. Mouse and human cytokine profiles was neither elevated nor down regulated, except for murine IL2p70. IL2p70 is a lymphocyte membrane glycoprotein (p70, IL-2RP) which aids IL-2 recruitment of lymphocytes and natural killer activity to the islet engraftment [51, 52]. The down regulation of IL2p70 may be potentially advantageous in dampening early immune responses. From these data, we interpret that MSCs are not harmful for *in vitro* islet survival and function, and the ability to downregulate pro-inflammatory cytokines could be dependent on *in vivo* function [3, 53].

Based on our *in vitro* observations, the 1:2000 islet to Ad-MSC ratio was used for *in vivo* transplantations due to their superior *in vitro* islet function. Without a culture period, we utilized a marginal mass dose of 150 islets, and attempted to transplant 150 islets post 48-hour culture, but due to the islet loss and subsequent decreased function, all mice transplanted maintained hyperglycemia and had to be euthanized prior to the end-point. We increased the dosage post culture to 200 islets per recipient to circumvent hyperglycemia to evaluate the islet engraftment for up to 60 days. Although islet mass transplanted was different, we have shown improved graft efficacy when islets were co-cultured with Ad-MSCs relative to islets co-transplanted with Ad-MSCs immediately after isolation without prior co-culture.

In this study, as a result of the islet loss in the control group compared to the islet and Ad-MSC group, the number of donor pancreata in the control group had to be increased to ensure that all groups were transplanted with a similar islet mass after 48-hour culture. We speculate improved graft function were due to Ad-MSCs formation and trophic effects during a prolonged direct cell contact of islets and Ad-MSC. The state of the Ad-MSCs prior to culture are single cells, and when transplanted MSCs could migrate away from the transplant site and therefore may not be as beneficial for engraftment. During a co-culture period, Ad-MSCs adhere to islets and one another and are more likely to remain at the engraftment site thereby increasing the probability for their trophic factors to help graft [54]. Extended culture times can reduce exocrine tissue and endotoxin carry-over [53] which may account for our observed marked improvement in islet potency after co-culture. We and others have demonstrated that MSCs can decrease islet loss, maintain islet morphology and function during co-culture with islets compared to culture of islets alone [24]. These results should be viewed with caution because early time points during *in vivo* assessment were not conducted and it is unclear whether MSCs benefits are derived only within the first days after engraftment, or have a prolong durable effect.

These findings are potentially readily translatable to the clinical setting, where human islets routinely undergo up to 72 hours of culture. Addition of human Ad-MSCs manufactured under good manufacturing conditions (GMP) during this period could be highly protective, and may decrease the need for multiple donors and risk of Human Leukocyte Antigen sensitization. Autologous MSCs offer potential promise in mitigating allo-rejection, perhaps allowing transplants to proceed with less immunosuppression and therefore less potential risk. To further elucidate the cytoprotective capacity of human Ad-MSCs our future research efforts will further explore the impact that co-culture on human islet function and engraftment.

Herein, we highlight a potential translatable strategy to protect islets in culture and early after transplantation that can increase the frequency of donor engraftment rates with the supplementation of Ad-MSC during culture.

## Supporting information

S1 Fig Murine and human proinflammatory cytokine analysis. Murine and human proinflammatory profiles from the medium after 48-hour culture (n = 3). Mouse cytokines are displayed on the left y-axis in blue and human cytokines on the right y-axis in red. Upon analysis, (**b**) murine IL2p70 expression was significantly downregulated for islets co-cultured with Ad-MSCs (1:2000) compared to control islets (*p<0.05, ANOVA) (mean ±s.e.m). IL-1β, IL-6, IL-10, and tumor necrosis factor (TNF)-α expression was detected, but no significance difference was demonstrated amongst groups (p>0.05, ANOVA). Data represented (mean ± s.d).
(TIFF)

S1 Table Kidney Islet Engraftment Insulin Content and Immunohistochemistry. Kidney bearing grafts were removed from mice ≥ 60 days post islet transplant (200 islets cultured 48-hours prior to transplant). Islet graft insulin content were similar for animals co-transplanted with islets with Ad-MSCs and islets alone (n *=* 3, p>0.05, t-test). Immunohistochemistry insulin and glucagon content in islet grafts were similar amongst islets transplanted with or without Ad-MSCs (200 islets, 1:2000 Ad-MSCs: islets alone n *=* 4 and Ad-MSCs n *=* 5) (p>0.05, t-test). Data represented (mean ± s.e.m).
(PDF)
