# Supplementary material for: Improved islet recovery and efficacy through co-culture and co-transplantation of islets with human adipose-derived mesenchymal stem cells
Source: PLoS One. 2018 Nov 12;13(11):e0206449. doi: 10.1371/journal.pone.0206449 (PMC6231609; doi:10.1371/journal.pone.0206449)
Supplement: S1 Table — Kidney bearing grafts were removed from mice ≥ 60 days post islet transplant (200 islets cultured 48-hours prior to transplant). Islet graft insulin content were similar for animals co-transplanted with islets with Ad-MSCs and islets alone (n = 3, p>0.05, t-test). Immunohistochemistry insulin and glucagon content in islet grafts were similar amongst islets transplanted with or without Ad-MSCs (200 islets, 1:2000 Ad-MSCs: islets alone n = 4 and Ad-MSCs n = 5) (p>0.05, t-test). Data represented (mean ± s.e.m). (PDF) [file pone.0206449.s002.pdf]

Cellular Component

Condition	Insulin Content (ng)	β %	α %
**Islets Alone (48-hour culture)**	2.07 ± 0.78	1.84 ± 0.93	1.38 ± 0.74
**Islets + 1:2000 Ad-MSCs (48-hour culture)**	3.07 ± 1.2	1.40 ± 1.08	1.42 ± 0.59
